# Structural Asymmetry of Phosphodiesterase-9, Potential Protonation of a Glutamic Acid, and Role of the Invariant Glutamine

**DOI:** 10.1371/journal.pone.0018092

**Published:** 2011-03-31

**Authors:** Jing Hou, Jie Xu, Ming Liu, Ruizhi Zhao, Hai-Bin Luo, Hengming Ke

**Affiliations:** 1 Structural Biology Lab, School of Pharmaceutical Sciences, Sun Yat-sen University, Guangzhou, People's Republic of China; 2 Department of Biochemistry and Biophysics and Lineberger Comprehensive Cancer Center, The University of North Carolina, Chapel Hill, North Carolina, United States of America; Institut Pasteur, France

## Abstract

PDE9 inhibitors show potential for treatment of diseases such as diabetes. To help with discovery of PDE9 inhibitors, we performed mutagenesis, kinetic, crystallographic, and molecular dynamics analyses on the active site residues of Gln453 and its stabilizing partner Glu406. The crystal structures of the PDE9 Q453E mutant (PDE9Q453E) in complex with inhibitors IBMX and (S)-BAY73-6691 showed asymmetric binding of the inhibitors in two subunits of the PDE9Q453E dimer and also the significant positional change of the M-loop at the active site. The kinetic analysis of the Q453E and E406A mutants suggested that the invariant glutamine is critical for binding of substrates and inhibitors, but is unlikely to play a key role in the differentiation between substrates of cGMP and cAMP. The molecular dynamics simulations suggest that residue Glu406 may be protonated and may thus explain the hydrogen bond distance between two side chain oxygens of Glu453 and Glu406 in the crystal structure of the PDE9Q453E mutant. The information from these studies may be useful for design of PDE9 inhibitors.

## Introduction

Cyclic nucleotide phosphodiesterases (PDEs) hydrolyze the second messengers cAMP and cGMP, and play crucial roles in many physiological processes. Twenty one of the human PDE genes encode about a hundred of PDE proteins that are categorized into eleven families on the basis of their biochemical and pharmacological properties [1]–[3]. PDE inhibitors have been widely studied as therapeutics for treatment of various diseases [4]–[9]. A well known example is the PDE5 selective inhibitor sildenafil that has been used for the treatment of male erectile dysfunction and pulmonary hypertension [4], [10]. Selective inhibitors of PDE9 have demonstrated potentials for treatment of human diseases, including insulin-resistance syndrome and diabetes [11], [12], cardiovascular diseases [13], obesity [14], and neurodegenerative disorders such as Alzheimer's disease [15]–[16].

PDE molecules contain an N-terminal regulatory domain and a conserved catalytic domain at the C-terminus. Individual PDE families display a preference for hydrolysis of the substrates cAMP (PDE4, 7, 8), cGMP (PDE5, 6, 9), or both (PDE1, 2, 3, 10, 11) [1]–[3], [17]. It has been a puzzle how the conserved active sites of PDEs selectively recognize the subtle differences between cAMP and cGMP. On the basis of the different conformations of the invariant glutamine in the crystal structures, a mechanism called “glutamine switch” was proposed for differentiation of the substrates by PDEs [18]. However, this hypothesis was challenged by the mutagenesis experiment [19] and the structural studies [20]–[22].

**Figure 1:**
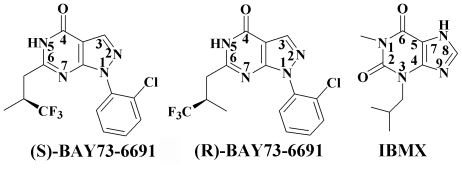
Chemical formulas of PDE9 inhibitors. 1-(2-chlorophenyl)-6-(3,3,3-trifluoro-2-methylpropyl)-1*H*-pyrazolo [3,4-*d*]pyrimidin-4(5*H*)-one (BAY73-6691) and 3-isobutyl-1-methylxanthine (IBMX).

To understand the roles of the invariant glutamine, we mutated Gln453 of PDE9A2 to glutamic acid (PDE9Q453E) and its stabilizing residue Glu406 to alanine, and measured the kinetic parameters of the mutants. In addition, we performed molecular dynamics (MD) simulations on the mutants and determined the crystal structures of PDE9Q453E in complex with the inhibitors 3-isobutyl-1-methylxanthine (IBMX) and (S)-BAY73-6691 (Fig. 1). Our studies reveal the structural asymmetry of PDE9 and potential protonation state of Glu406, and also suggest that Gln453 is unlikely to play a key role in differentiation of the substrates.

## Materials and Methods

### Molecular cloning, site-directed mutagenesis, and protein expression

The catalytic domain of the wild type human PDE9A2 (GenBank number BC009047) was subcloned to vector pET15b by following the protocol described previously [23]. The coding region for residues 181-506 of PDE9A2 was amplified by PCR. The amplified PDE9A2 cDNA and the expression vector pET15b were digested by the restriction enzymes NdeI and XhoI, purified by agarose gel electrophoresis, and then ligated together by T4 DNA ligase. The recombinant plasmid was selected by ampicillin resistance and verified by DNA sequencing (Sangon, China). A site-directed mutagenesis kit (Stratagene, USA) was used to generate the PDE9A2 mutants of Q453E and E406A. The methylated parental plasmid was digested by DpnI endonuclease. The mutations were verified by DNA sequencing.

The wild type and mutants of the PDE9A2 catalytic domain were purified by using the similar protocols previously reported [24]. In brief, the recombinant plasmid was transferred into *E. coli* strain BL21 (Codonplus, Stratagene). The *E. coli* cells carrying the pET-PDE9A2 plasmids were grown in LB medium at 37°C to absorption A600 = ∼0.7 and then 0.1 mM isopropyl β-D-thiogalactopyranoside was added to induce expression. The cells after induction were grown at 15°C overnight. Recombinant PDE9A2 proteins were purified by column chromatography of Ni-NTA affinity (Qiagen), Q-Sepharose ion-exchanging (GE Healthcare), and Sephacryl S300 gel filtration (GE Healthcare). A typical batch of purification yielded 20–100 mg PDE9A2 from a 2-liter cell culture. The PDE9A2 proteins had purity greater than 95%, as shown by SDS-PAGE.

### Enzymatic assay

The enzymatic activities of the wild type PDE9A2 and its mutants were assayed by using cAMP and cGMP as substrates. A 100 µl reaction mixture contained 50 mM Tris-HCl pH 8.2, 10 mM MgCl_2_, 0.5 mM DTT, 174 nM ^3^H-cAMP or 30 nM ^3^H-cGMP (30,000–100,000 cpm, GE Healthcare), and various concentrations of cAMP or cGMP. Each measurement was repeated two times. The reaction was carried out at room temperature for 15 min and then terminated by the addition of 0.2 M ZnSO_4_ and 0.2 M Ba(OH)_2_. The reaction product ^3^H-AMP or ^3^H-GMP was precipitated by BaSO_4_, whereas unreacted ^3^H-cAMP or ^3^H-cGMP remained in the supernatant. Radioactivity in the supernatant was measured in 2.5 ml Ultima Gold liquid scintillation cocktails (PerkinElmer) by a PerkinElmer 2910 liquid scintillation counter. Vmax and K_M_ values were calculated by nonlinear regression on the curve of velocity versus substrate concentration and also by Eadie-Hofstee plot. For the measurement of IC_50_ of inhibitors, nine concentrations of inhibitors, 30 nM ^3^H-cGMP, and the enzyme concentration that hydrolyzed up to 70% of the substrate were used. The inhibitors IBMX, (S)-BAY73-6691, and zaprinast were purchased from Sigma-Aldrich. The IC_50_ values were calculated by nonlinear regression.

### Crystallization and structure determination

The catalytic domain of the PDE9Q453E mutant (10–15 mg/mL, amino acids 181–506) was stored in a buffer of 50 mM NaCl, 20 mM Tris.HCl pH 7.5, 1 mM β-mercaptoethanol, and 1 mM EDTA. After mixing with 2 mM IBMX, the PDE9Q453E-IBMX complex was crystallized by hanging drop vapor diffusion against the well buffer of 2.0 M Na formate, 0.1 M HEPES pH 7.5, 5% xylitol at 4°C. Crystals of the PDE9Q453E-(S)-BAY73-6691 complex were prepared by soaking PDE9Q453E-IBMX co-crystals in the crystallization buffer plus 2 mM (S)-BAY73-6691 at 25°C for 3 days. The crystals were flash-frozen in liquid nitrogen by using the well buffer containing saturated xylitol as the cryosolvent. X-ray diffraction data were collected at 100 K on Beamline BL17U of Shanghai Synchrotron Radiation Facility, China (Table 1) and processed by HKL2000 [25]. The structures of the PDE9Q453E mutant in complex with IBMX and (S)-BAY73-6691 were solved by the molecular replacement program AMoRe [26], using the PDE9A2 catalytic domain [23] as the initial model. The atomic model was rebuilt by program O [27] or COOT [28] against the electron density maps that were improved by the density modification package of CCP4. The structures were refined by CNS [29] and REFMAC [30]. The atomic coordinates and structure factors have been deposited into the Protein Data Bank with accession numbers of 3QI3 and 3QI4.

**Table 1:** Statistics on diffraction data and structure refinement.

*Data collection*	PDE9A2(Q453E)-(S)-BAY73-6691	PDE9A2(Q453E)-IBMX
Space group	P4_1_2_1_2	P4_1_2_1_2
Unit cell (*a*, *c*, Å)	103.1, 270.4	103.4, 270.0
Resolution (Å)	2.3	2.5
Total measurements	382,636	665,895
Unique reflections	63,252	50,388
Completeness (%)	95.8 (67.3)*	97.6 (72.5)*
Average I/σ	14.5 (2.3)*	12.2 (6.2)*
Rmerge	0.092 (0.54)*	0.094 (0.24)*
*Structure Refinement*		
R-factor	0.222	0.213
R-free	0.242 (10.0%)‡	0.239 (10.0%)‡
Resolution (Å)	15-2.3	15-2.5
Reflections	58,317	48,879
RMS deviation for		
Bond (Å)	0.007	0.007
Angle	1.2°	1.2°
Average B-factor (Å)		
Protein	49.3 (5372)§	39.9 (5372)
Inhibitor	64.1 (48)	49.5 (48)
Zn	61.0 (2)	61.9 (2)
Mg	46.2 (2)	45.5 (2)
Water	43.8 (23)	42.1 (29)

*The numbers in parentheses are for the highest resolution shell.

‡The percentage of reflections omitted for calculation of R-free.

§The number of atoms in the crystallographic asymmetric unit.

### Molecular dynamics simulations

The crystal structures of the wild type PDE9A2 in complex with (R)- or (S)-BAY73-6691 (PDB access codes of 3K3E and 3K3H) [24] and of the Q453E mutant in complex with (S)-BAY73-6691 (this study) were used to generate the PDE9A2 monomers for the MD simulations. The hydrogen atoms were added by software Sybyl 7.3.5. The protocol of the restricted electrostatic potential fitting, as implemented in the Antechamber module of the AMBER 10 package [31], was used for calculations of the partial atomic charges of (R)- or (S)-BAY73-6691 at the *ab initio* HF/6-31G* level. The Zn^2+^ and Mg^2+^ ions were treated by the non-bonded method [32]. The bridging ligand between the two metal ions was set as HO^−^, as previously proposed [33]. The parameters of the AMBER general force field (GAFF) and ff03 were used for PDE9A2 and (R)-/(S)-BAY73-6691 [34]. The PDE9A2 complexes were neutralized by adding sodium counter ions and solvated with water molecules within 10 Å radius to protein atoms.

The structures were first minimized for 6000 steps to remove possible steric stress. The relaxed structures were gradually heated from 0 to 300 K in 100 ps increments and then equilibrated for 200 ps at 300 K using the NVT (number of particles, volume, and temperature) ensemble. Weak constraints of 10 and 2 kcal • mol^−1^ • Å^−2^ were applied on the proteins during the heating and equilibrating procedures, respectively. Finally, periodic boundary dynamics simulations of 8 ns were carried out by using the NPT (number of particles, pressure, and temperature) ensemble at 1 atm and 300 K. The SHAKE algorithm [35] was turned on for the covalent bonds involved in hydrogen atoms with a tolerance of 1×10^−5^ Å. The simple harmonic motion was applied to other covalent bonds. The Particle-Mesh-Ewald method [36] was applied to treat the long range electrostatic interactions with a 10 Å non-bonded cutoff.

## Results

### Subtle conformational changes in the PDE9Q453E structures

**Figure 2:**
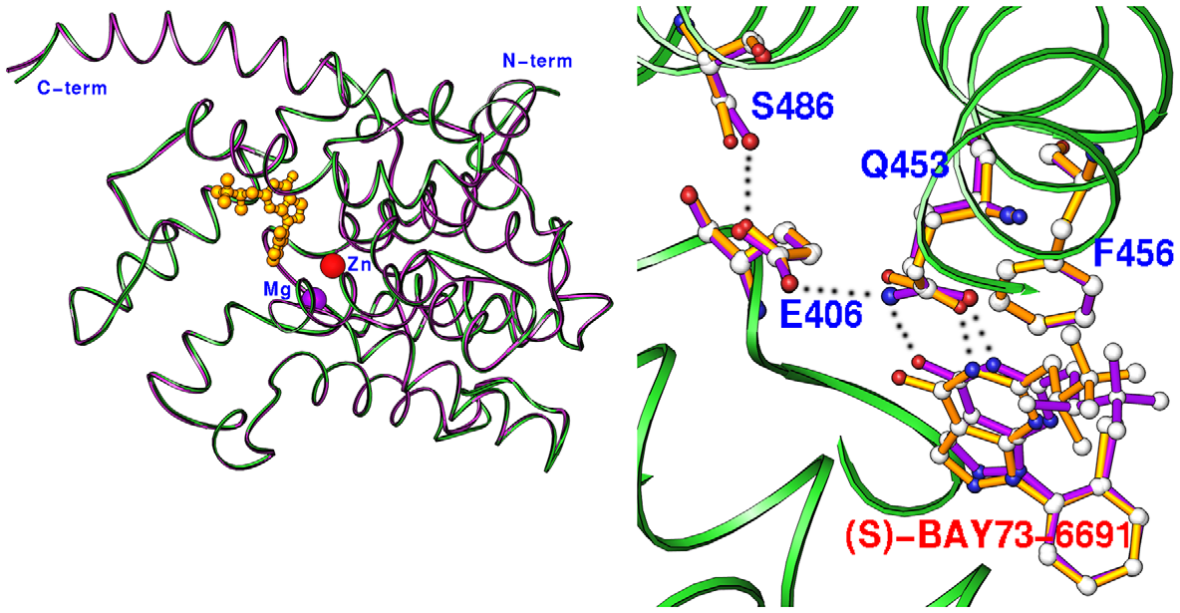
Structures of the PDE9Q453E mutant in complex with (S)-BAY73-6691. (A) Ribbon presentation of superposition of the wild type PDE9A2 (green) over the PDE5Q453E mutant (purple) in complex with (S)-BAY73-6691 (golden balls). (B) Conformational change of Glu453 in the PDE9Q453E mutant. The side chain of Glu453 rotated by about 15° to relieve the repulsion between electron-rich oxygens of Glu453 and (S)-BAY73-6691. The residues of the wild type PDE9A2 and the PDE9Q453E mutant are presented in purple and golden colors, respectively.

The crystallographic asymmetric unit of the PDE9Q453E mutant in complex with IBMX or (S)-BAY73-6691 contains two molecules of the PDE9A2 catalytic domain. A monomer of the PDE9Q453E mutant is composed of 16 helices (Fig. 2A) that are folded into a similar topology as PDE9 and other PDE families [17]. The Q453E mutation did not significantly change the overall structure, as shown by the small root-mean squared deviations (RMSD) of 0.17 and 0.28 Å for the superposition of Cα atoms of the corresponding chains (A over A and B over B) between the mutant and the wild type enzyme [23], [24]. However, the side chain of Glu453 showed significant conformational change due to the mutation. In the wild type PDE9A2, the side chain of Gln453 was stabilized by a hydrogen bond (2.8 Å) between NE2 of Gln453 and OE1 of Glu406. In the Q453E mutant structure, the side chain of Glu453 rotated about 15° (Fig. 2B). This movement made a distance of 3.8 Å from OE2 of Glu453 to O_4_ of (S)-BAY73-6691, and 3.2 Å to OE1 of Glu406. The 3.2 Å distance between two electron-rich oxygen atoms may cause energetically unfavored repulsion. A possible explanation may be that Glu406 is protonated, as discussed in the section on MD simulations.

### Asymmetry of the inhibitor binding and the PDE9 dimer

**Figure 3:**
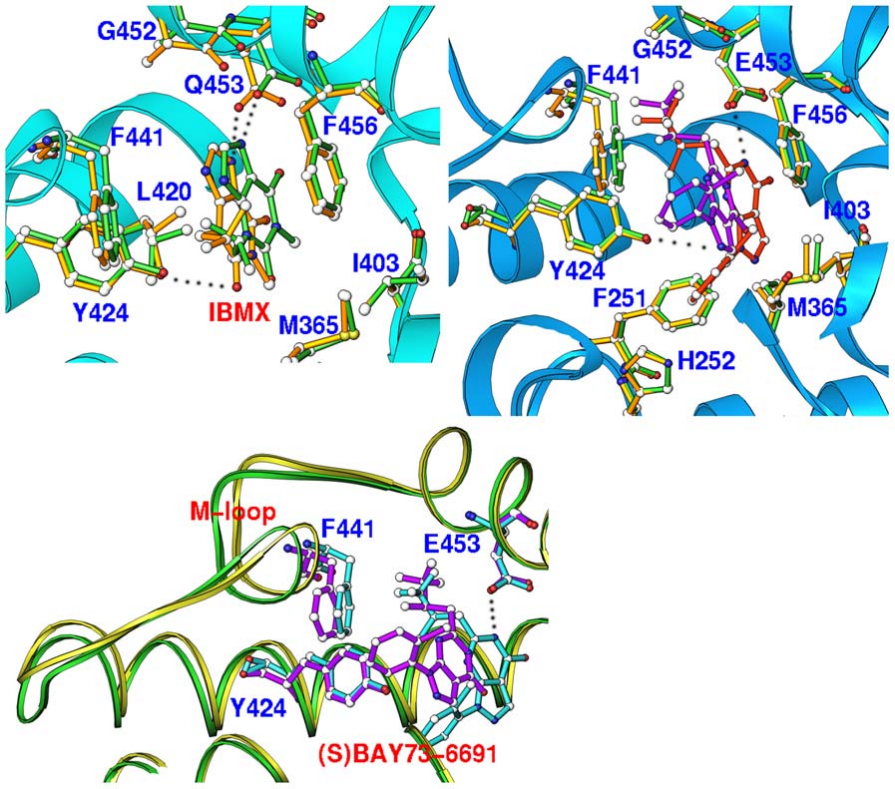
Asymmetric binding of the inhibitors to the active site of the PDE9Q453E mutant. (A) Interactions of IBMX with the residues in subunit A (green sticks) and subunit B (golden). IBMX forms an additional hydrogen bond (dotted lines) with Tyr424 in subunit B, but not in subunit A. (B) Superposition of the interactions of (S)-BAY73-6691 with the residues in subunit A (green and red sticks) and subunit B (golden and purple). (C) Positional changes of the M-loop at the active site of the PDE9Q453E mutant. Subunit A is shown in cyan and golden colors while green and salmon are for subunit B.

The major force for the IBMX binding to the active site of the PDE9Q453E mutant was the hydrogen bond between N7 of IBMX and OE1 of Glu453 and the stack of the xanthine ring of IBMX against Phe456 (Fig. 3A). This feature is conserved in both chains A and B and is similar to the IBMX binding in the wild type PDE9A2. However, IBMX binding showed certain characteristics of asymmetry in chains A and B of the PDE9Q453E dimer. First, the conformation of IBMX was defined by the clear electron density in chain B, but was less definite in chain A (Fig. S1). The B-factors were 58 and 41 Å^2^, respectively for IBMXs in chains A and B, indicating relatively lower occupancy of IBMX in chain A. Second, two IBMXs showed a positional difference of about one Angstrom, as revealed by the superposition between chains A and B, in spite of the conservation of their stack against Phe456 and interaction with the common PDE9A2 residues (Fig. 3A). Finally, the N3 atom of IBMX in chain B formed a hydrogen bond with OH of Tyr424 (3.2 Å), whereas the same interaction in chain A shows a distance of 3.6 Å, which belongs to a typical polar interaction (Fig. 3A).

The asymmetry is also observed in the binding of inhibitor (S)-BAY73-6691 to the PDE9Q453E mutant. In the PDE9Q453E-(S)-BAY73-6691 structure, the conformation and position of (S)-BAY73-6691 to chain A was clearly defined by the electron density maps of (2Fo - Fc) and (Fo - Fc) (Fig. S1). However, the electron density in chain B had the quality only to reveal the binding location but not the accurate conformation of the inhibitor. The B-factor of (S)-BAY73-6691 in chain A (56 Å^2^) is significantly smaller than that in chain B (72 Å^2^), consistent with the observation of low occupancy of (S)-BAY73-6691 in china B. The interaction of (S)-BAY73-6691 with chains A and B in the PDE9Q453E dimer is very different. In chain A, the pyrazolo-pyrimidine ring of (S)-BAY73-6691 stacked against the phenyl ring of Phe456 and its N5 atom formed a hydrogen bond with OE1 of Glu453 (Fig. 3B). In chain B, (S)-BAY73-6691 formed no hydrogen bond with Glu453, but with Tyr424 (Fig. 3B), although its pyrazolo-pyrimidine ring remained stacked against Phe456.

While the biological implication of the asymmetric binding of IBMX and (S)-BAY73-6691 in the PDE9Q453E structures is unclear, it appears to be the consequence of the structural asymmetry of two subunits in the PDE9Q453E dimer. Indeed, the structural superposition between chains A and B yielded RMSDs of 0.64 and 0.77 Å, respectively for the structures of PDE9Q453E-IBMX and PDE9Q453E-(S)-BAY73-6691. These values are 2–3 times the RMSDs resulting from the comparison of the same subunits between the wild type PDE9 and its mutants (A versus A and B versus B). Detailed examination showed significant positional changes around residues 440–450 that have differences 2 to 3 times the overall average for the Cα atoms (Fig. 3C). This fragment is known as the M-loop in the PDE families, which is a component of the active site and has been implicated to play important roles in catalysis [37]. Since the M-loop also shows the positional differences in the structures of the wild type PDE9A in complex with substrate cGMP or inhibitors [22]–[24], the asymmetry might have implications for design of PDE9 inhibitors.

### The impact of Q453E and E406A mutations on the enzymatic properties

The PDE9Q453E mutant had the K_M_ values of 2.8 µM for cGMP and 1.2 mM for cAMP, which represent about 25- and 2.5-fold loss in comparison with the apparent affinity of the wild type enzyme [23]. The catalytic activities of the PDE9Q453E mutant toward cGMP and cAMP were also reduced to about half of those of the wild type enzyme (Table 2). Thus, the Q453E mutation caused a loss of about 50- and 5-folds of enzymatic efficacy, k_cat_/K_M_, for cGMP and cAMP, respectively. The reduction of the enzymatic efficacy for cGMP might be interpreted by the fact that the Q453E mutation abolishes the hydrogen bond between NE2 of Gln453 and O6 of cGMP. However, the interpretation of the loss of cAMP activity is unclear because the Q453E mutation would be predicted not to change the number of hydrogen bond with cAMP and thus the activity.

**Table 2:** Kinetic parameters of the catalytic domain of PDE9A2 and its mutants.

Enzymes	cGMP			cAMP			(k_cat_/K_M_)^cGMP^/(k_cat_/K_M_)^cAMP^
	K_M_ (µM)	Vmax(µmol/mg/min)	k_cat_(S^−1^)	K_M_ (µM)	Vmax(µmol/mg/min)	k_cat_(S^−1^)	
Wild type	0.113±0.013	0.285±0.008	0.18±0.00	501±43	3.70±0.10	2.37±0.06	337
Q453E	2.8±0.18	0.139±0.003	0.09±0.00	1233±92	2.03±0.07	1.30±0.04	29
E406A	0.311±0.04	0.129±0.004	0.08±0.00	338±44	0.48±0.02	0.31±0.01	280

The E406A mutation reduced the apparent affinity and the catalytic activity of cGMP by only 2-fold (Table 2). The E406A mutation did not significantly change the affinity for cAMP, but decreased the catalytic activity by about 8-fold (Table 2). In the crystal structure of the wild type PDE9A2, Glu406 stabilizes the side chain conformation of Gln453 with a hydrogen bond [22]–[24]. The E406A mutation will abolish this hydrogen bond and thus presumably allow the side chain of Gln453 to rotate. As a result, cAMP may form two hydrogen bonds with the side chain of Gln453 after the rotation and the E406A mutant would thus be expected to have increased cAMP activity. However, no significant change on the apparent affinity (K_M_) for cAMP, but reduction of k_cat_ might imply that the Gln453 side chain does not rotate in the E406A mutant. Thus, our kinetic study provides new evidence against the mechanism of the “glutamine switch”, in which Gln453 is assumed to rotate for the recognition of substrate cGMP or cAMP.

Regarding the inhibitor binding, the Q453E mutation led to a minor loss in the affinity of IBMX and zaprinast (Table 3), but showed a radical impact on (S)-BAY73-6691. The IC_50_ value of (S)-BAY73-6691 for the PDE9Q453E mutant was >160 µM, which is >1800 fold to IC_50_ of 86 nM for the wild type enzyme. A possible interpretation to the different affinity of the inhibitors might be ascribed to the binding modes among these inhibitors. The non-selective and weak inhibitors IBMX and zaprinast are relatively small and may adopt different orientations in the large PDE9 binding pocket to result in a similar affinity in both wild type and mutant enzymes. However, since the selective PDE9 inhibitor (S)-BAY73-6691 fits tightly to the binding pocket, the Q453E mutation would cause a net loss of the hydrogen bond and thus impact on the binding affinity. On the other hand, the E406A mutation did not significantly alter the affinity of the three inhibitors (Table 3). This may be understandable because Glu406 does not directly interact with the inhibitors.

**Table 3:** Effects of the mutations on IC_50_ of inhibitors.

Enzymes	IBMX (µM)	(S)-BAY73-6691 (nM)	Zaprinast (µM)
Wild type	53.9±1.1	85.8±1.0	15.9±1.2
Q453E	117.2±1.2	(164.2±1.2)×10^3^	53.7±1.2
E406A	31.7±1.1	82±1.1	7.6±1.1

### Glu406 is likely protonated in the PDE9Q453E structure as shown by MD simulations

To further understand the role of Gln453, we performed MD simulations on the mutants PDE9Q453E and PDE9E406A. Initially, the known structures of the wild type PDE9A2 in complex with (R)-/(S)-BAY73-6691 were used to tune the program for MD simulations. After a 4 ns MD run, stable trajectories with RMSD of <2 Å were obtained. The average distances from NE2 and OE1 of Gln453 to O_4_ and N5 of (S)-BAY73-6691 are 2.9 and 2.8 Å in the MD model (Fig. S2), which compares well with the distances of 2.9 and 2.7 Å in the crystal structure. In addition, the pyrazolo-pyrimidine ring of (S)-BAY73-6691 remained stacked against Phe456 in the entire MD trajectories. Since the hydrogen bond with the invariant glutamine and the stack against phenylalanine are two key characteristics of inhibitor binding in almost all PDE structures [17], the MD simulations apparently simulate the crystal structures well.

**Figure 4:**
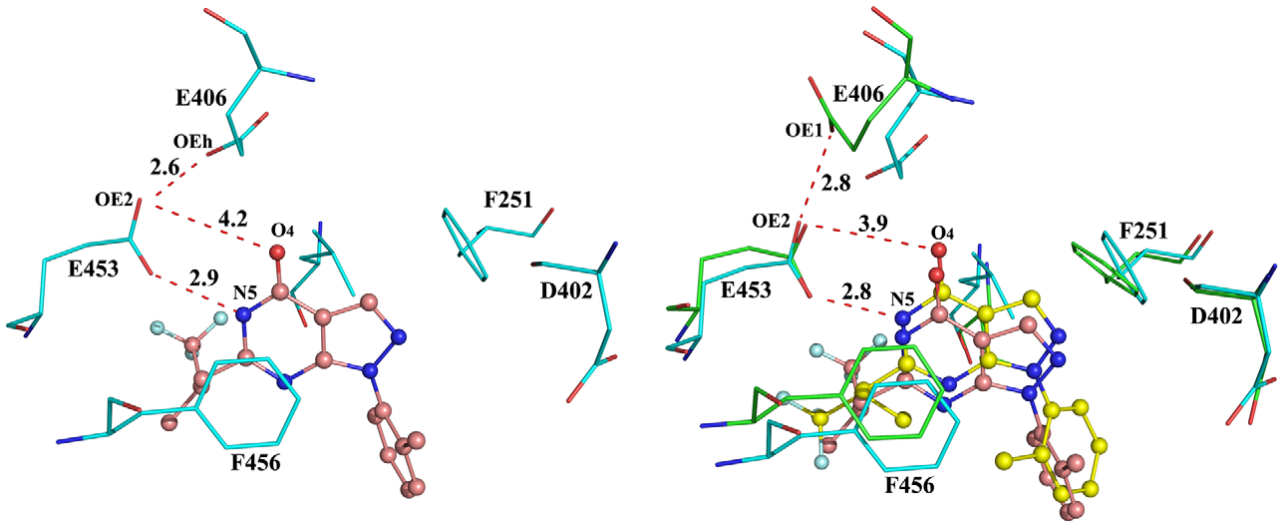
Simulated binding of (S)-BAY73-6691 at the active site of the PDE9Q453E mutant. (A) Interactions in the MD simulation model. The carboxyl group of Glu406 was treated as a protonated form (marked as OEh). (B) Superposition of the crystal structure of PDE9Q453E-(S)-BAY73-6691 (green and yellow) over the MD simulation model (cyan and salmon). The marked distances come from the crystal structure.

Under the same conditions, MD simulations on the PDE9Q453E-BAY73-6691 complex reached equilibration with an average RMSD of 2.1 Å. In the simulated model, the pyrazolo-pyrimidine ring of (S)-BAY73-6691 stacked against Phe456, in agreement with that of the crystal structure. However, two key distances between OE1 of Glu453 and N5 of (S)-BAY73-6691, and between OE2 of Glu453 and OE1 of Glu406 were 5.3 and 4.2 Å, respectively, in contrast to 2.9 and 3.2 Å in the crystal structure of the PDE9Q453E mutant. Since 3.2 Å represents the distance of a hydrogen bond and no proton is expected to associate with OE2 of Glu453 and OE1 of Glu406 under the crystallization pH 7.5, a possible assumption might be that Glu406 in the PDE9Q453E mutant locally sequesters a hydrogen atom and thus exists in the protonated form. To test this hypothesis, a new model with protonated Glu406 was subjected to MD simulations under the same conditions (Fig. S2). In this MD simulation model, the pyrazolo-pyrimidine ring of (S)-BAY73-6691 stacks against Phe456 (Fig. 4); atom OE2 of Glu453 is in a hydrogen bond distance (2.6 Å) to the protonated OE1 of Glu406 and 4.2 Å to O_4_ of (S)-BAY73-6691; atom OE1of Glu453 forms a hydrogen bond (2.9 Å) with N5 of (S)-BAY73-6691. Therefore, the new MD simulations agree well with the crystal structure and suggest that Glu406 in the crystal of the PDE9Q453E mutant likely exists in its protonated form.

## Discussion

### The “glutamine switch” is unlikely the mechanism for differentiation of substrates

An essential question about the PDE function is how PDE molecules distinguish the two alternate substrates cAMP and cGMP that show very subtle differences. An early proposal on the substrate specificity, the “glutamine switch”, is based on the crystal structures of PDE4 and PDE5 in complex with their corresponding reaction products [18]. In these structures, the side chain of the invariant glutamine takes opposite orientations to form two hydrogen bonds with AMP or GMP. The “glutamine switch” proposal assumes that the invariant glutamine in the dual specific PDE families switches its side chain conformations to gain two hydrogen bonds with different substrates. However, this hypothesis was challenged by the observations that substrates cAMP and cGMP have different orientations and interactions in the structures of the dual specific PDE10 [20], and that cAMP forms only one, but not two hydrogen bonds with cAMP-specific PDE4 [21]. In addition, the Q817A mutation in PDE5A reduces the cGMP affinity by about 60-fold, but does not affect the cAMP kinetic behavior [19]. Since the replacement of hydrophilic glutamine with hydrophobic alanine may cause relatively large changes, we performed the Q453E mutation to eliminate the effect of the side chain rotation of Gln453.

Our studies provide further evidence against the “glutamine switch” mechanism. First, on the basis of the crystal structure of PDE9-cGMP [22], the Q453E mutation would be predicted to reduce the cGMP activity, but not to impact the cAMP activity because the Q453E mutation will lead to loss of a hydrogen bond with cGMP but no change with cAMP. However, our kinetic data showed a 4.5-fold loss of the cAMP catalytic efficacy by the Q453E mutant (Table 2), implying that substrate recognition is not achieved by the simple rotation of the glutamine side chain. Second, the E406A mutation would allow the Gln453 side chain to rotate and to potentially form two hydrogen bonds with cAMP. Thus, the E406A mutation would be expected to increase the catalytic efficacy. However, the kinetic data of the E406A mutant showed that the K_M_ for cAMP essentially remained at the same level and k_cat_ decreased significantly. Therefore, our data suggest that the “glutamine switch” is unlikely to be the mechanism for differentiation of the substrates, although the invariant glutamine is critical for binding of substrates and inhibitors, as shown by 25- and 1800-fold affinity decrease of the mutant for cGMP and (S)-BAY73-6691, respectively.

### Conclusions

1. The binding of inhibitors IBMX and (S)-BAY73-6691 to the PDE9Q453E mutant shows significant asymmetry and the M-loop demonstrates the significant positional difference in the dimer of the PDE9Q453E mutant.
2. The kinetics of the Q453E and E406A mutants suggest that the side chain of Gln453 may not rotate and thus the “glutamine switch” is unlikely to be the mechanism for the substrate recognition by PDEs.
3. The MD simulations suggest that Glu406 may be protonated in the PDE9Q453E mutant and thus is capable of forming a hydrogen bond with Glu453. This may explain the unusual proximity of two negatively charged oxygen atoms in the crystal structure.

## Supporting Information

Figure S1 **Electron density for (A) IBMX in subunit A of the PDE9AQ453E mutant, (B) IBMX in subunit B, (C) Bay73-6691 in subunit A, and (D) Bay73-6691 in subunit B.** The (Fo – Fc) maps were calculated from the structures in which the inhibitors were omitted, and contoured at 2.5 sigmas.
(TIF)

Figure S2 **Variation of the key non-bonded distances during simulation.** (A) Distance change between O_4_ of (S)-BAY73-6691 and NE2 of Gln453 (black), and N5 of (S)-BAY73-6691 and OE1 of Gln453 (red) in the wildtype PDE9A2. (B) Distance change between O_4_ of (S)-BAY73-6691 and OE2 of Glu453 (black), N5 of (S)-BAY73-6691 and OE1 of Glu453 (red), and OE2 of Glu453 and protonated OE1 (labeled as OEh) of Glu406 (blue) in the PDE9Q453E mutant.
(TIF)
