# Supplementary figures and images for: Structural Asymmetry of Phosphodiesterase-9, Potential Protonation of a Glutamic Acid, and Role of the Invariant Glutamine
Source: PLoS One. 2011 Mar 31;6(3):e18092. doi: 10.1371/journal.pone.0018092 (PMC3069055; doi:10.1371/journal.pone.0018092)

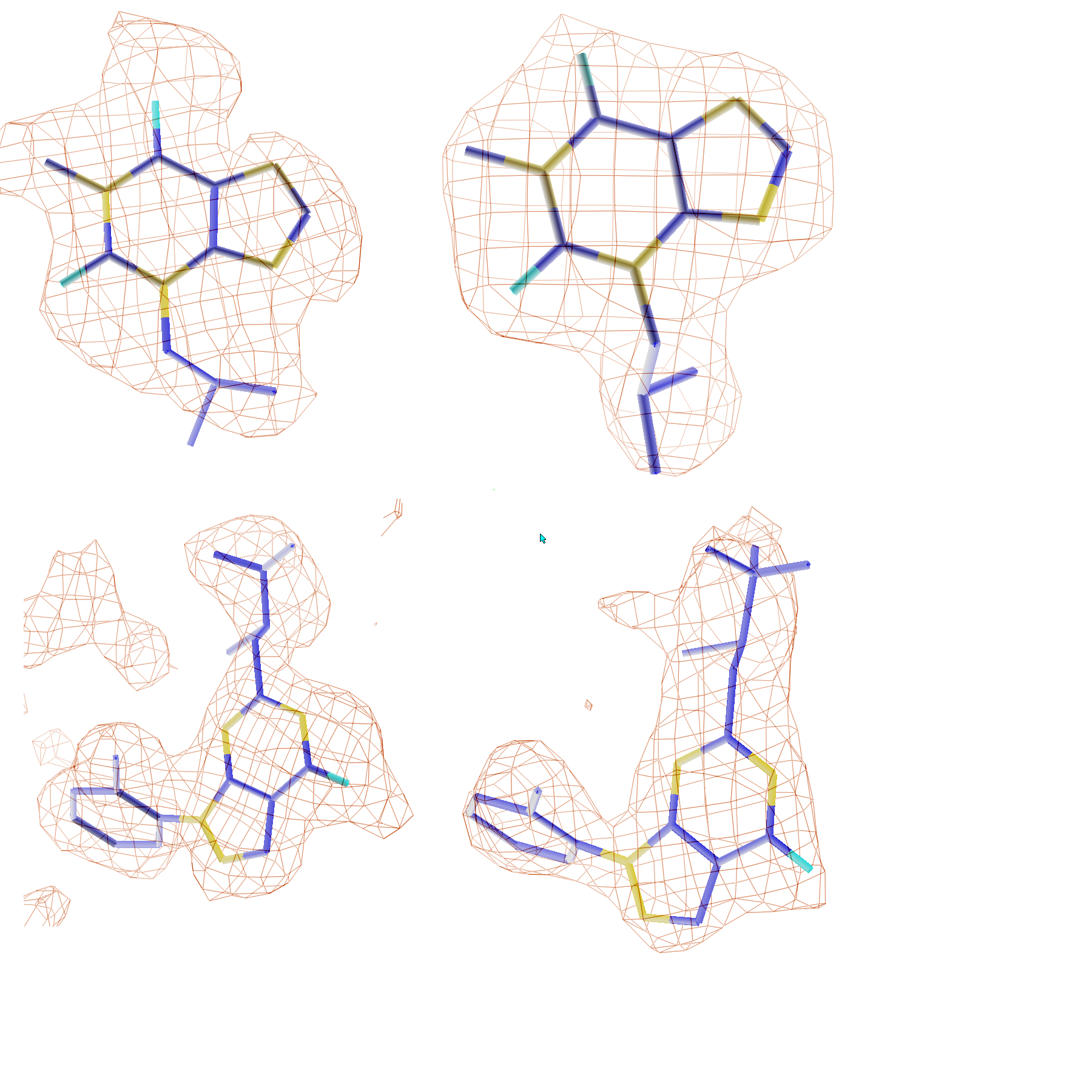

Supplement: Figure S1 — Electron density for (A) IBMX in subunit A of the PDE9AQ453E mutant, (B) IBMX in subunit B, (C) Bay73-6691 in subunit A, and (D) Bay73-6691 in subunit B. The (Fo – Fc) maps were calculated from the structures in which the inhibitors were omitted, and contoured at 2.5 sigmas. (TIF) [file pone.0018092.s001.tif]
